# Spatial genetic characterization of the red fox (Vulpes vulpes) in the area between the Alps and the Central Dinaric Mountains

**DOI:** 10.18699/vjgb-24-83

**Published:** 2024-11

**Authors:** A. Devedžić, F. Urzi, B. Pokorny, G. Vengušt, D.Ž. Vengušt, F. Janžekovič, L. Velić, T. Eterović, B.K. Stroil, E. Bužan

**Affiliations:** Faculty of Natural Sciences and Mathematics, University of Maribor, Maribor, Slovenia; Faculty of Mathematics, Natural Sciences and Information Technologies, University of Primorska, Koper, Slovenia; Faculty of Environmental Protection, Velenje, Slovenia; Veterinary Faculty, University of Ljubljana, Ljubljana, Slovenia; Veterinary Faculty, University of Ljubljana, Ljubljana, Slovenia; Faculty of Natural Sciences and Mathematics, University of Maribor, Maribor, Slovenia; Faculty of Veterinary Medicine, University of Sarajevo, Sarajevo, Bosnia and Herzegovina; Faculty of Veterinary Medicine, University of Sarajevo, Sarajevo, Bosnia and Herzegovina; Institute of Genetic Engineering and Biotechnology, University of Sarajevo, Sarajevo, Bosnia and Herzegovina; Faculty of Mathematics, Natural Sciences and Information Technologies, University of Primorska, Koper, Slovenia Faculty of Environmental Protection, Velenje, Slovenia

**Keywords:** red fox, Vulpes vulpes, microsatellites, genetic diversity, Dinaric Mountains, красная лисица, Vulpes vulpes, микросателлиты, генетическое разнообразие, Динарские горы

## Abstract

Red fox, Vulpes vulpes, is a globally distributed species characterized by its high adaptability to diverse habitats and a broad range of food resources. This remarkable adaptability has allowed the red fox to thrive in various environments, from urban areas to remote wilderness. In this study, we used a set of microsatellite markers for the comparative genetic analysis of red fox populations from two countries. We included populations from the Eastern Alps and the northern Dinaric Mountains in Slovenia, as well as the Central Dinaric Mountains in Bosnia and Herzegovina. We successfully isolated DNA and genotyped 118 red fox samples. Our analyses, which included Bayesian clustering techniques, revealed a weak genetic differentiation among the studied populations. However, it is noteworthy that statistically significant differences in estimates of genetic differentiation were only apparent when comparing the populations between the two countries. Further spatial genetic clustering analyses provided additional insights, unveiling a differentiation into four genetic clusters. These clusters comprised two distinct groups in Bosnia and Herzegovina and two in Slovenia. This pattern of differentiation suggests that isolation by distance is a key factor influencing the genetic structure of the red fox in this studied region. Additionally, our findings highlighted that populations from the Alps and northern Dinaric Mountains exhibit higher genetic diversity and observed heterozygosity compared to their counterparts in the Central Dinaric Mountains. The genetic diversity is also notable when compared to other European red fox populations. Studying genetic diversity is crucial for the resilience and adaptability of populations, ensuring their survival amid environmental changes and human-induced pressures.

## Abstract (ru)

Красная лисица, Vulpes vulpes, – широко распространенный вид, характеризующийся высокой адаптивностью к различным средам обитания и широким спектром пищевых ресурсов. Удивительная адаптивность позволила красной лисице выживать в самых разнообразных условиях, от городских территорий до отдаленных диких мест. С использованием набора микросателлитных маркеров нами проведен сравнительный генетический анализ популяций красной лисицы из двух стран. В исследование включены популяции из Восточных Альп и северных Динарских гор в Словении, а также из центральных Динарских гор в Боснии и Герцеговине. Мы успешно выделили ДНК и генотипировали 118 образцов красной лисицы. Анализы, включавшие байесовские методы кластеризации, показали слабую генетическую дифференциацию между исследуемыми популяциями. Однако надо отметить, что статистически значимые различия в оценках генетической дифференциации были очевидны лишь при сравнении популяций между двумя странами. Дополнительные пространственные генетические анализы выявили разделение на четыре генетических кластера, которые включали две отдельные группы в Боснии и Герцеговине и две – в Словении. Такая картина дифференциации предполагает, что ключевым фактором, влияющим на генетическую структуру красной лисицы в данном регионе, является изоляция по расстоянию. Кроме того, наши результаты показали, что популяции из Альп и северных Динарских гор обладают более высокими генетическим разнообразием и наблюдаемой гетерозиготностью по сравнению с популяциями из центральных Динарских гор. Генетическое разнообразие заметно также при сравнении с другими европейскими популяциями красной лисицы. Изучение генетического разнообразия имеет важное значение для устойчивости и адаптируемости популяций, обеспечивая их выживание в условиях экологических изменений и антропогенного давления.

## Introduction

Red fox (Vulpes vulpes) is recognized as one of the most widespread terrestrial predators in Europe, potentially ranking among the most prevalent mammals (Hoffmann et al., 2004). Its extensive distribution across diverse European environments exposes the species to a spectrum of environmental and climatic conditions, contributing to variations in life strategies and fitness among populations (Nowak, 1999). Red fox exhibits adaptability across various habitats, including forests, tundra, prairies, deserts, mountains, agricultural lands, and urban areas. It is often regarded as a pest due to its perceived detrimental effects on prey populations and its capacity to transmit various diseases. In many European countries, it is considered an important game species (Atterby et al., 2015).

The species is native both in Slovenia and Bosnia and Herzegovina (B&H). In Slovenia, red fox is the most common and predominant wild carnivore, and populations are widespread from the Adriatic coast to the Prekmurje region. In the period 2014–2023, between 10,055 (in 2014) and 14,707 (in 2021) individuals were hunted annually, and reported roadkill in the same period was in the range from 686 (in 2023) to 1,177 (in 2019) individuals, respectively. Moreover, every year between approx. 200 and 300 red foxes were found dead either due to diseases (mainly sarcoptic mange) or unknown reasons (OSLIS, 2024). In B&H, red fox occupies a variety of habitats with an estimated density of around 0.5 individuals per km2. Considering hunting data, it is estimated that about 24,000 foxes live in B&H, distributed over the entire country (Nemet, 2018).

Red fox populations exhibit pronounced lack of mitochondrial DNA (mtDNA) genetic structuring on a wide spatiotemporal scale (Frati et al., 1998; Teacher et al., 2011; Edwards et al., 2012; Kutschera et al., 2013; McDevitt et al., 2022). The lack of phylogeographic structuring in red fox, based on mtDNA markers, has been attributed to its persistence outside the traditional refugia areas during the last glacial maximum (LGM), but several lines of evidence suggest survival of red fox phylogenetic lineages in southern refugia (Kutschera et al., 2013). Southern European regions generally have less species connectivity than northern ones, but populations still show low differentiation (Frati et al., 1998; Kirschning et al., 2007; Gachot-Neveu et al., 2009; Edwards et al., 2012). Other studies based on nuclear DNA markers confirmed low genetic diversity of red foxes in Europe, including Poland (Mullins et al., 2014), United Kingdom (Atterby et al., 2015), and Scandinavia (Norén et al., 2015). These studies also revealed significant genetic structure at smaller geographic scales, suggesting the formation of distinct subpopulations within regions.

Red fox genetic diversity, structure, and gene flow between populations are influenced by internal factors such as vagility and dispersion, and external factors like landscape and environment. Landscape features, such as rivers or mountain ranges, may restrict gene flow and impact genetic diversity of subpopulations (Manel et al., 2003; Kirschning et al., 2007; Valvo, 2011; Sommer S. et al., 2013; Galov et al., 2014; Balkenhol et al., 2015). Historical and current factors, including past population bottlenecks, range expansions, landscape features, and habitat fragmentation, collectively influence red fox population genetic diversity and structure (Frati et al., 1998; Teacher et al., 2011; Edwards et al., 2012; Kutschera et al., 2013; Statham et al., 2014; McDevitt et al., 2022).

Two geographically wider and comparative studies (Zecchin et al., 2019; McDevitt et al., 2022) have already studied the genetic structure of red foxes in Slovenia. Zecchin et al. (2019) revealed that red foxes in Slovenia belong to a unified group together with the Croatian population, and that there is no spatially and temporally extensive phylogeographic structure of the species within Europe. Similarly, McDevitt et al. (2022) showed that the Slovenian red fox population belongs to the “Central Europe” cluster together with populations from Croatia and Serbia. Both studies used nuclear markers (microsatellites and single nucleotide polymorphisms, respectively) and underlined the importance of genomic data in identification of refugia regions and post-glacial expansions across Europe (e. g. the Balkans), while at the same time providing necessary insights on red fox genetic diversity and structure

Red fox from Croatia was individually studied by Galov et al. (2014). They found a high degree of genetic diversity among individuals, indicating a wide range of genetic variation within the population. Despite mitochondrial haplotype diversity, which is among the highest of all European red fox populations, the study showed a remarkable lack of population structure, indicating extensive gene flow and interbreeding of red foxes in the country. The study by Kirschning et al. (2007) included red foxes from Serbia, and different mitochondrial haplotypes were found, demonstrating genetic structuring within the population. However, as with the neighbouring population from Croatia (Galov et al., 2014), a relatively low degree of genetic differentiation was found on nuclear genetic markers between different geographical regions within Serbia.

To date, there has been no research on the genetic structure of red fox in Bosnia and Herzegovina. Therefore, the primary aim of our study was to conduct an analysis of the genetic variability and structure of red fox populations in B&H in comparison with the Slovenian population. More broadly, we also attempted to establish conclusions regarding the connections with neighbouring populations from Croatia and Serbia.

## Materials and methods

Study area and sampling. The study area included territories of B&H and Slovenia (Supplementary Material 11), covering an area of around 66,189 km2 (Nemet, 2018; Hunting Association of Slovenia, 2021).


Supplementary Materials are available in the online version of the paper: https://vavilovj-icg.ru/download/pict-2024-28/appx25.pdf


Animals used in the study were either legally harvested during the hunting season or collected as roadkill or natural death. No animal was shot or otherwise killed for the purposes of this study solely. Samples of harvested animals were collected by hunters or wildlife researchers immediately after harvest between 2019 and 2021. Tissue samples were preserved in 70 % ethanol and blood samples were stored at –20 °C until analysis. In line with our objectives, samples included in the analysis were collected in Slovenia (n = 59) and B&H (n = 59). The Faculty of Veterinary Medicine, University of Sarajevo and the Veterinary Faculty, University of Ljubljana, which oversee the rabies surveillance initiative in both countries, sent only negative samples for genetic analysis at the Molecular Ecology Laboratory at the University of Primorska.

DNA extraction and quality control. The extraction of DNA from collected samples was performed using the peqGOLD Blood & Tissue DNA Mini Kit (VWR International, LLC, Austria), following the manufacturer’s instructions. The concentration and purity of DNA obtained were measured with a 3.0 Qubit Fluorometer using Invitrogen™ – Qubit™ dsDNA BR Assay Kit (Life Technologies, Carlsbad, CA, USA). Nineteen microsatellites (Supplementary Material 2), originally identified and screened in canine genome studies in red foxes and domestic dogs (Richman et al., 2001; Kukekova et al., 2004, 2007), were amplified in three multiplex PCR reactions with DNA Thermo Cycler (Applied Biosystems). Amplification was carried out with ready-to-use KAPA2G Fast Multiplex Mix (Kapa Biosystems) in 15 μl of the reaction mixture containing 5 μl of template DNA (~25 ng DNA), and 0.2 mM final concentration for each primer used in the set. The amplification was performed under the following conditions: initial denaturation step at 95 °C for 3 minutes, followed by 30 cycles of denaturation for 35 seconds, annealing 1 Supplementary Materials 1–6 are available at: https://vavilovj-icg.ru/download/pict-2024-28/appx25.pdf at 58 °C for 35 seconds, extension at 72 °C for 35 seconds; a final extension step at 72 °C for 10 minutes.

The fragment analysis was performed on a SeqStudio sequencer (Thermo Fisher Scientific) using the GeneScan LIZ500 (−250) standard (Applied Biosystems). The results were validated with the software GENEMAPPER v.5.0 (Applied Biosystems). Null alleles and heterozygosity deficiency were assessed using FreeNA (Chapuis, Estoup, 2007), a program based on Dempster’s algorithm, to avoid Hardy–Weinberg equilibrium (HWE) deviation. GENEPOP 4.7 software (Rousset, 2008) was utilised to perform the exact test for heterozygosity deficiency, calculating the deviation from HWE and inbreeding coefficient (FIS) estimates. Statistical significance was set at p < 0.05

Genetic diversity parameters, including the number and richness of alleles, were computed using GENETIX 4.05.2 (Belkhir et al., 2004), FSTAT 2.9.4 (Goudet, 1995), and GENEPOP 4.7 (Rousset, 2008). Pairwise fixation index (FST) between populations was assessed using the Weir and Cockerham algorithm (1984) in GENEPOP 4.7, employing 1,000 permutations for statistical rigour.

STRUCTURE 2.3.4 (Falush et al., 2003) was employed to determine population structure, assessing the probability of genetic interbreeding between individuals. The model considered the unique origin of each ancestor for a specific allele, providing the probability (Q) that each subject belongs to a particular cluster or group. For STRUCTURE, 10 independent cycles for each K (number of clusters) were conducted between 1 and 10, using a Markov chain model with 1,000,000 Markov Chain Model Monte Carlo (MCMC) iterations and 100,000 burn-in iterations for each cycle. We applied a mixed model featuring independent allele frequencies. Depending on the specific populations under investigation, we opted for the independent allele frequencies model, if the allele frequencies among distinct populations differ accordingly. The program STRUCTURE Harvester v0.6.94 (Earl, Vonholdt, 2014) was used to combine results and determine the most optimal K based on ΔK developed by Evanno et al. (2005), with results recorded using CLUMPP (Jakobsson, Rosenberg, 2007) and DISTRUCT (Rosenberg, 2004).

Discriminant analysis of principal components (DAPC) was employed to identify genetic clusters within the dataset using the Adegenet package (Jombart, 2008) in R 3.5.1 software (R Development Core Team, 2011). DAPC, a multivariate statistical method, reduces genetic data dimensionality through principal component analysis (PCA) and uses discriminant analysis to identify genetic clusters or subpopulations

For spatial population structure analysis, a visual user interface (GUI) was developed in the R environment using Geneland 4.9.2 (Guillot et al., 2005). Geneland, a Bayesian method, employs MCMC simulations to estimate the number of genetic populations (K) and assigns individuals to populations based on genetic similarity, considering spatial autocorrelation and isolation by distance

Arlequin ver. 3.5.2.2 (Excoffier, Lischer, 2010) was used for the analysis of molecular variance (AMOVA), providing techniques to test the genetic differences between individuals and populations and between the optimal number of clusters identified by STRUCTURE (K = 2). Finally, we tested isolation by distance (IBD) patterns within all genetic populations

through the Mantel test, comparing genetic and geographical distances. Euclidean distances were calculated using R 3.5.1 software (R Development Core Team, 2011) and the Adegenet library (Jombart, 2008). Significance was determined through 999 Monte Carlo simulations, evaluating the correlation between Edwards distances and Euclidean geographic distances

## Results

We visualised fox genotypes with GeneMapper v.5.0 software. Six monomorphic markers and six markers with high null allele frequencies (>20 %) were excluded. Monomorphic markers lack variation in DNA sequence length, while null alleles result in non-amplification of expected alleles in PCR tests. We than proceeded with statistical analyses on the remaining seven markers (V402, Vv-C01.424, VVM189, Vv- REN169O18, FH2541, Vv-INU055, and Vv-C08.618). The purpose of these exclusions is to ensure the accuracy of the results and prevent any unfair impact on the genetic variability parameters of the populations under study.

Genetic diversity within B&H and Slovenian red fox populations

Our findings revealed lower genetic diversity in the B&H population, i. e. evident in lower observed heterozygosity and allelic richness (HO = 0.648, AR = 7.538) compared to the Slovenian population (HO = 0.770, AR = 9.724). The genetic diversity in B&H was also lower than genetic diversity of red fox across Austria, Croatia, Italy, and Slovenia (HO = 0.75, AR = 12.6) as reported by Zecchin et al. (2019). However, this difference could be a result of a higher number of microsatellites used in Zecchin et al. study, which is three times higher than in the present one. Conversely, compared to the analysed Slovenian population, the observed heterozygosity was lower but allelic richness was higher in this regional study of red foxes. The observed heterozygosity in B&H foxes was lower than observed heterozygosity of foxes from Poland (HO = 0.729, AR = 4.871), even though they used only microsatellite markers (Mullins et al., 2014). In contrast, the Slovenian population displayed higher HO and AR values compared to Polish population. Finally, both B&H and Slovenian populations had greater observed heterozygosity and allelic richness than red foxes from the United Kingdom (Atterby et al., 2015; 11 microsatellite loci: HO = 0.543, AR = 5.033).

Genetic differentiation between B&H and Slovenian red foxes

**Fig. 1.**
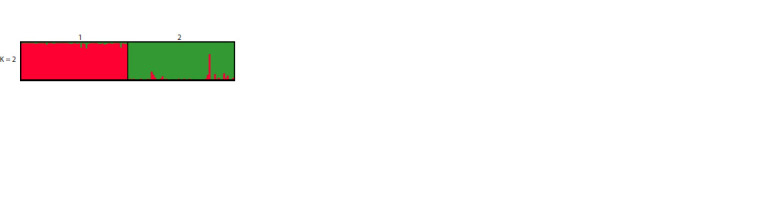
Genetic structure of red fox populations in Slovenia (1) and B&H (2), determined by STRUCTURE Each individual is represented by a line proportionally partitioned into colour segments corresponding to its membership in particular cluster. K is the number of clusters

**Fig. 2.**
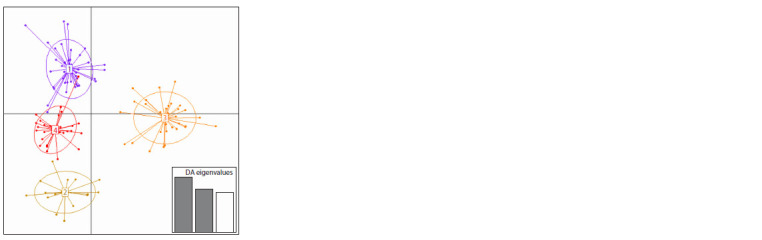
The DAPC scatter plot shows the presence of four clusters (K = 4). Cluster 1 includes red fox samples from Slovenia, while the Cluster 3 includes red fox samples from B&H. In the Clusters 2 and 4 we can find samples from both countries (Supplementary Material 5).

Spatial genetic structure and genetic clustering depend on the software and analysis employed. For instance, STRUCTURE (Fig. 1) and Geneland identified two clusters (K = 2): one includes red fox from B&H and the other one samples from Slovenia (Supplementary Materials 3 & 4). On the contrary, DAPC analysis suggested four clusters (K = 4) (Fig. 2), dividing each countries’ populations into two additional clusters. These discrepancies stem from different methodologies and assumptions inherent to each approach in population genetics analysis.

**Fig. 3.**
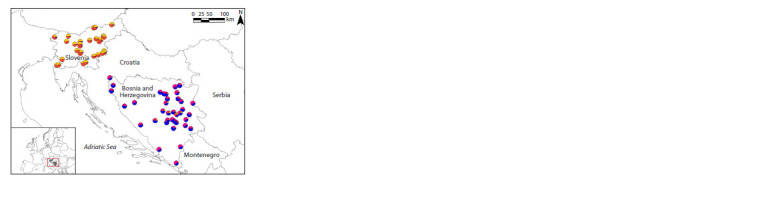
Genetic structure of red fox in two studied countries based on the CLUMPP Q values and harvesting locations (K = 4 clusters). Q values represent the proportion of individuals that are assigned to each cluster shown by GIS mapping (Supplementary Material 6).

STRUCTURE estimates ancestry proportions and admixture based on genotypes, relying on assumptions like linkage equilibrium and HWE. DAPC employs dimensionality reduction and discriminant analysis for membership assignment, emphasising differences between clusters. Geneland combines spatial and genetic clustering. All three methods confirmed spatial differentiation at the regional level, with a weaker structure or admixture identified by STRUCTURE and DAPC, possibly due to long-distance dispersal of red foxes. The assumption of HWE was violated in STRUCTURE and Geneland analyses, affecting quantitative estimates. However, due to geographic or ecological constraints, there may be subpopulations with limited gene flow, which geographic information system (GIS) image (Fig. 3) clearly revealed by combining our microsatellite data and sampling coordinates in both populations.

DAPC revealed that red foxes from Slovenia form a distinct cluster (Cluster 1), while Clusters 2, 3, and 4 are shared between both countries. Cluster 3, predominant in B&H individuals, displayed the most genetic variation. Cluster 2 was genetically closer to B&H, and Cluster 4 showed high similarity to Cluster 1, suggesting potential admixture between B&H and Slovenian red fox populations. Historical gene flow from Slovenia to the Balkans was supported by previous studies, aligning with post glacial colonisation patterns (Sommer R.S., Nadachowski, 2006).

AMOVA indicated genetic differentiation among populations and substantial within-individual variation. The FST value of 0.068 suggested significant but not highly differentiated populations, with 6.9 % of genetic variation attributed to differences between populations ( p < 0.001).

IBD analysis demonstrated a positive correlation ( p <0.001, R2 = 0.13; this correlation was higher than the randomly simulated p-value of 0.001) between genetic and geographic distances for B&H and Slovenian red fox populations, indicating differentiation due to limited dispersal, landscape barriers, habitat fragmentation, resource distribution, interactions with other species or human activities. However, we acknowledge the influence of factors like distance between B&H and Slovenia (almost 300 km). Understanding how habitat changes affect mobile species like red fox, especially in terms of genetic structure, remains an on-going challenge. Landscape history, barriers to movement, and migration rates can leave lasting genetic patterns and impact current population structures. Overall, our findings emphasise the importance of considering diverse analytical approaches and acknowledging the influence of geographical factors on genetic structure in red fox populations.

## Discussion

We successfully revealed genetic structuring of the red fox populations between Alps and the Central Dinaric Mts. and showed weak genetic differentiation of the species in the studied area. Our findings are consistent with previous ones, which revealed the presence of a genetically unified red fox population in Slovenia (Zecchin et al., 2019; McDevitt et al., 2022). The DAPC analysis divided the Slovenian red foxes into two clusters, indicating the existence of possible subpopulations and admixture between the North Dinaric Mts. and the Prekmurje region, but this could also be a consequence of isolation by distance. Red foxes from B&H also belong to one STRUCTURE cluster, but the DAPC analysis divided them into two genetic clusters: first including the area of the Central Dinaric Mts. and second in the northern parts of the country. The observed genetic structure by DAPC analysis can be attributed to various factors. The territory of B&H contains significant natural and anthropogenic barriers, such as mountain ranges and rivers, potentially leading to population isolation and genetic drift effects. The Dinaric Mts. along the western border with Croatia as well as internal mountain ranges may limit gene flow between Croatia and B&H. Galov et al. (2014) demonstrated the limiting effects of Istrian narrow land bridge and mountain altitudes above 1,000 m on fox migration in Croatia, which can also be the case for areas in B&H and Slovenia with many topographical similarities in studied habitats.

Although our results suggest limited gene flow between Slovenian and B&H red fox populations, the absence of Croatian samples hinders relevant conclusions. However, Zecchin et al. (2019) found a unified genetic structure of the fox in Croatia and Slovenia. Despite this, we can assume that there are some possible barriers to gene flow among foxes in the studied regions such as rivers like Sava, a tributary of the Danube, which forms the northern border between B&H and Croatia, and the Drina, which flows north and forms part of the eastern border between B&H and Serbia. The influence of rivers on red fox movement was also discussed in the population genetic analysis of Serbian red foxes by Kirschning et al. (2007). The study highlighted the role of the two major rivers, the Danube and the Tisza, which flow through Serbia, as potential barriers to fox migration. There are also anthropogenic barriers that lead to genetic isolation of foxes in B&H, such as the highway that crosses Croatia and runs through the north-western part of the border between B&H and Croatia.

The existence of two genetic groups of red fox between Alps and northern Dinaric Mts. and other in the Central Dinaric Mts. was previously showed by Zecchin et al. (2019), who identified separated clusters in Friuli Venezia Giulia region as a border area in which circulating individuals are genetically more like those from Slovenia and Croatia than to those of the remaining areas of north-eastern Italy. As a support to our results, a phylogenetic analysis of the mitochondrial cytochrome b and D-loop by Statham et al. (2014) identified a clear differentiation between Italian red foxes and the red fox populations from the Balkans and Eastern Europe. Our results indicated that the B&H population belongs to the Balkan cluster according to mtDNA but additional analysis is needed to confirm this assumption.

The limited number of genetic markers used in our study likely influenced the Bayesian genetic structure analysis, resulting in an inability to distinctly separate subgroups by STRUCTURE and Geneland analysis (Falush et al., 2003). This limitation becomes more evident when compared to the clearer subgroup differentiation observed in the DAPC analysis. On the other hand, the landscape barriers such as rivers and lower mountains might not restrict gene flow among foxes in Slovenia, Croatia, and B&H. Indeed, in the studied area there is adequate habitat connectivity for red fox, therefore it seems that landscape characteristics do not cause important barriers to gene flow, resulting in lack of population differentiation in this species (Kirschning et al., 2007). This can also be confirmed by weak phylogeographic structuring of red fox on much larger geographic scales, such as within the Holarctic lineage (Kutschera et al., 2013).

Despite Galov et al. (2014) identified significant mtDNA genetic structure of red fox on the Istrian Peninsula, which borders coastal Slovenia, our data did not reveal similar genetic differentiation in the Slovenian population. This could likely be due to stable connectivity with neighbouring Italian population and the rest of the Slovenian population. However, it might also be due to variety of markers used in both studies.

Red foxes in Bosnia and Herzegovina have lower genetic diversity comparing to Slovenian and other European populations, which could be a consequence of a recent population decline due to rabies epidemics, compared to Slovenia and Croatia, where oral vaccination programmes (ORV) of foxes led to the control of the rabies epidemic (Rabies Bulletin Europe, 2017). Indeed, since the implementation of the ORV campaign in Slovenia (in 1988) and Croatia (in 2011), the number of rabies cases have been decreasing consistently with no cases detected since 2015 (Bedeković et al., 2016; Picot et al., 2017). The last rabies case in Slovenia was recorded in 2013, whereas in B&H was present until 2020. There, the lack of economic resources has led to a lack of continuous ORV, resulting in continued rabies hotspots in B&H (Tasioudi et al., 2014; Lojkić et al., 2021). Non-suitable disease management may lead to decrease in population size and influence the genetic diversity in the B&H red fox population, both in terms of allelic richness and observed heterozygosity compared to Slovenian population.

The statistically significant p-values for the FIS estimates in Slovenia and B&H red fox population ( p = 0.37 and p = 0.13, respectively) can be associated with historical bottlenecks due to diseases but also with possible regional isolation of populations (which was not revealed in our study, possible due to limited number of markers), leading to lower observed heterozygosity

The results of AMOVA suggested existing genetic differences among populations. High genetic variation among individuals within sampled populations suggests substantial gene flow between them. However, the high level of genetic diversity within populations may suggest that maintaining habitat connectivity is an important factor in promoting gene flow and maintaining genetic variation within populations. These values demonstrate statistically significant differentiation between observed locations; however, it is crucial to acknowledge that the interpretation can vary based on factors like the particular genetic markers employed, the sampling strategy adopted, and the genetic structure inherent in the populations.

The significant IBD values among populations indicate a pattern of isolation by distance, attributed to missing data from neighbouring populations in Croatia. Assuming that the distance between the closest locations in the two countries exceeds several hundred kilometres, the observed genetic divergence is probably consequence of an isolation by distance. Considering also the DAPC analysis, which also confirmed shared genetic makeup between populations despite the distance, as has also been demonstrated in a study from Poland (Mullins et al., 2014).

Our findings are consistent with those of Teacher et al. (2011), who reported a small degree of isolation by distance, in mitochondrial control region, and a wide-scale absence of phylogeographic structure based on cytochrome b data, in red foxes in Western Europe. The species’ versatility and capacity to adapt to a broad range of habitats, along with a comparatively high degree of dispersal in both males and females, are suggested to be the main reasons for the low level of genetic structure observed (Teacher et al., 2011).

## Conclusion

This study enhanced our understanding of the red fox genetic structure in the region encompassing the Eastern Alps and the Dinaric Mountains. In alignment with previous research, we found the absence of a pronounced genetic structure within the red fox population in this area. Populations from Slovenia and B&H showed low level of genetic differentiation, with significant differences found only when comparing populations in countries.

## Conflict of interest

The authors declare no conflict of interest.
